# Image Registration for Visualizing Magnetic Flux Leakage Testing under Different Orientations of Magnetization

**DOI:** 10.3390/e25010167

**Published:** 2023-01-13

**Authors:** Shengping Li, Jie Zhang, Gaofei Liu, Nanhui Chen, Lulu Tian, Libing Bai, Cong Chen

**Affiliations:** 1 School of Automation Engineering, University of Electronic Science and Technology of China, Chengdu 611731, China; 2 China Petroleum Pipeline Insptection Technologies Co., Ltd., Langfang 065000, China

**Keywords:** nondestructive testing, Magnetic Flux Leakage, solenoid modal, image registration, mutual imformation, PSO

## Abstract

The Magnetic Flux Leakage (MFL) visualization technique is widely used in the surface defect inspection of ferromagnetic materials. However, the information of the images detected through the MFL method is incomplete when the defect (especially for the cracks) is complex, and some information would be lost when magnetized unidirectionally. Then, the multidirectional magnetization method is proposed to fuse the images detected under different magnetization orientations. It causes a critical problem: the existing image registration methods cannot be applied to align the images because the images are different when detected under different magnetization orientations. This study presents a novel image registration method for MFL visualization to solve this problem. In order to evaluate the registration, and to fuse the information detected in different directions, the mutual information between the reference image and the MFL image calculated by the forward model is designed as a measure. Furthermore, Particle Swarm Optimization (PSO) is used to optimize the registration process. The comparative experimental results demonstrate that this method has a higher registration accuracy for the MFL images of complex cracks than the existing methods.

## 1. Introduction

**Figure 1:**
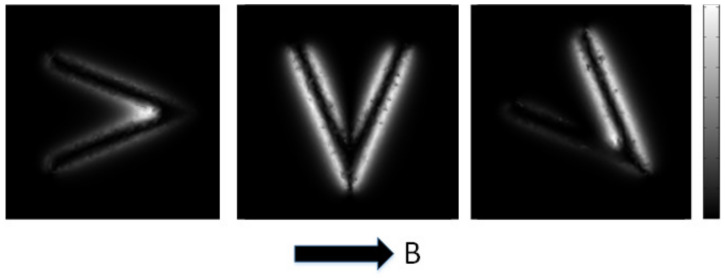
The distribution of MFL field with DOM (Simulated by COMSOL).

Magnetic Flux Leakage (MFL) detection is widely used in the nondestructive testing of defects in ferromagnetic components [1,2,3,4]. The inversion for the profile of a defect is the most interesting part of the related research. In existing studies of 3D profiles, the effect of the surface profile of the defect on the defect depth is often not considered [5,6]. However, the surface profile of the defect severely affects the distribution of the MFL field in space. Existing profile reconstruction methods mainly use data collected using magnetization in a single direction [2,7], which is effective when there are no edges that are parallel to the magnetization direction, or complex corners in the defects. However, cracks are prone to more complex signal coupling, or they are missing due to their small width and complex surface profile, which cannot be collected completely from a single direction, and they must be magnetized from multiple directions to obtain complete information about the surface profile of the defect [8,9]. (Figure 1 shows the different MFL field distributions of a V-shaped defect under different orientations of magnetization.) Traditional MFL testing needs to scan the same area several times in different directions. In order to analyze the MFL signal characteristics of the acquired defects, a method is required first to align the acquired MFL images under different orientations of magnetization (DOM).

Image registration is defined as aligning images acquired at different times, distinct viewpoints, and where valuable information is conveyed in more than one image [10]. The MFL images captured under DOM are difficult to spatially align in actual application because of the displacement and rotation of sensors. However, no published papers discuss the MFL image registration of defects captured under DOM, to the authors’ knowledge.

The general image registration methods (e.g., medical image registration and remote sensing image registration) are mainly classified as being intensity-based and feature-based [11]. Intensity-based methods use optimization methods to find the optimal value of the alignment. Different pixel intensity-based measures are applied to evaluate the registration of each iteration of the optimization process [12,13]. However, such methods cannot consider the inconsistency of the magnetic field distribution under DOM. They can only match the parts of the image with similar intensity distributions, because such methods are based on the assumption that the corresponding structures in the registered images would have similar intensities [10]. Additionally, the MFL field distribution detected under DOM varies greatly, making the shape alignment methods inapplicable [14].

The feature-based approaches use distance-based measures to match the features extracted from the input images. The methods require the presence of features: such as centerlines, outlines, corners, etc. Additionally, the images must possess a relatively clear corresponding point mapping [13,15,16], which would also be affected by the distortion of MFL because the distribution of MFL images varies (such as the first and the second image in Figure 1).

**Figure 2:**
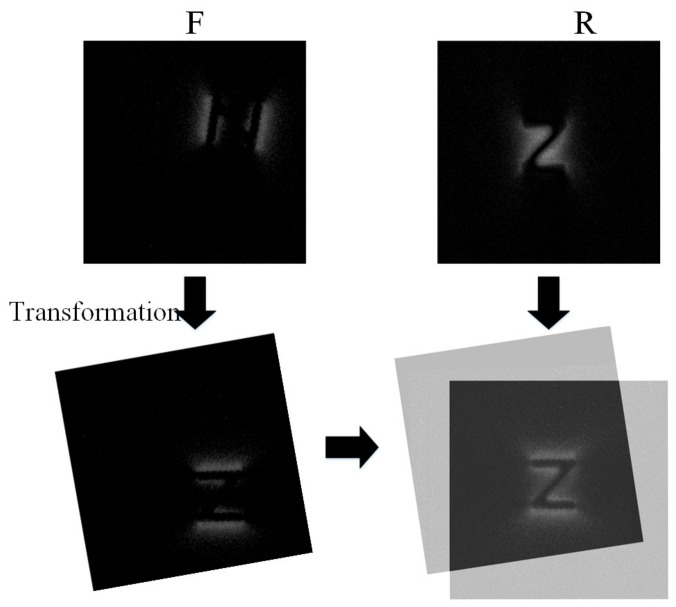
Registration Schematic of MFL images under DOM.

The above analysis shows that the currently used image registration methods do not apply to the MFL images captured under DOM. This paper proposes an adaptive registration method to register the multidirectional magnetized MFL images of surface defects (as in Figure 2). The proposed method combines the MFL forward model and the multi-model image alignment method. Firstly, the PSO optimization method updates the image transform parameters and generates a new defect shape according to the aligned images. Then, the MFL field distribution of the new reconstructed shape is generated via the use of the solenoid model [8,17]. The similarity between the generated MFL field distribution and the acquired reference image is calculated as the judgment of optimization. Using the above process, the optimal alignment parameters are achieved, and the registration of the MFL images under DOM is completed.

There are three contributions to this paper:

1. A new registration method for MFL images detected under DOM is designed.
2. The solenoid model is first used in MFL image registration.
3. The comparative experiment is carried out, and the proposed method shows a higher accuracy than the traditional methods.

This paper is organized as follows. Section 2: The proposed method is introduced. Section 3: The experimental setup. Section 4: Presents the results and discussion. Section 5: The conclusions.

## 2. Methodology

Most conventional registration methods can be described as an optimization process. During each step of the optimization algorithm, a new parameter of the transformation function is updated, which is applied to the floating image (*F*) by spatially aligning it with the reference image (*R*). Additionally, the registration performance is accessed using a measure of similarity between the reference and the transformed floating image. The procession is shown in Figure 2.

**Figure 3:**
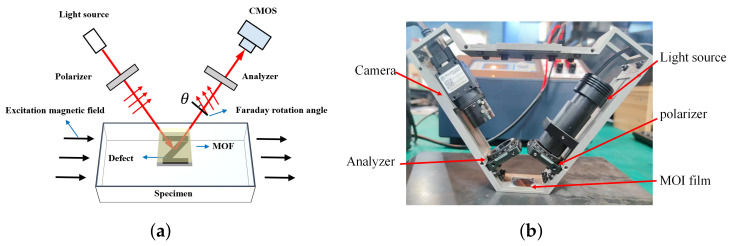
Magneto-optical imaging: (**a**) Schematic of MOI, (**b**) The MOI system.

Herein, the reference image (*R*) and floating image (*F*) are captured by the magneto-optical image (MOI) system, which is shown in Figure 3. The polarized light passes through the magneto-optical film (MOF), which rotates due to the Faraday rotation effect, and the rotation angle θ can be calculated by Equation (1). The rotated light reflected by a mirror under the MO film would be filtered by the polarizer and captured by the CMOS. The acquired gray image depicts the normal components of the MFL field.

(1) $$\begin{array}{c} \theta =VBL \end{array}$$

where *V* is the Verdet constant of the MOF, and *B* is the intensity of the introduced magnetic field parallel to the direction of the light. *L* denotes the distance that the light travels through the MOF.

**Figure 4:**
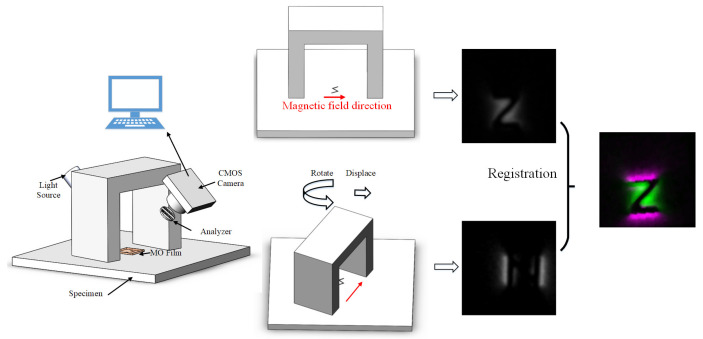
Schematic of the procession.

The MFL image detection under DOM can be expressed as in Figure 4. The angle between the MOI system and the magnetic yoke is fixed to keep the direction of the external magnetic field in the captured images. Additionally, the performance of registration would be better when the rotation angle comes to the position where the images are complementary. (Complete information about defects can be obtained at an angle of about 80${}^{\circ }$∼90${}^{\circ }$).

**Figure 5:**
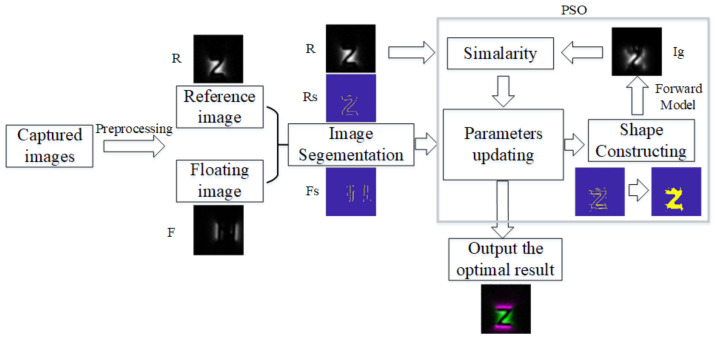
The proposed method.

Like the prevailing algorithms, the proposed registration algorithm consists of an iterative trial-and-error process that attempts to optimize a given transformation function after a limited number of iterations [16,18]. The process of the proposed registration method can be described, as shown in Figure 5. The first step is in segmenting the original images *R* and *F* to $R_{s}$ and $F_{s}$. Then, Particle Swarm Optimization (PSO) is used to maximize the similarity by optimizing the transformation of in-plane parameters. In each iteration of PSO, five steps are needed:

1. Producing a new registration parameter;
2. Transforming the $F_{s}$ according to the registration parameter and aligning it to $R_{s}$;
3. Fusing the $R_{s}$ and the transformed $F_{s}$, then reconstructing a shape of crack ($I_{t}$);
4. Generating a new distribution ($I_{g}$) of the crack ($I_{t}$);
5. Calculating the similarity between *R* and $I_{g}$.

After the iterations, the parameter of the optimal similarity is output as the final result of registration.

### 2.1. Preprocessing

A rough registration to limit the translation is needed to reduce the computation time and to avoid the registration parameters escaping from the feasible solution space (a high similarity may occur, even if the images are not correctly registered).

**Figure 6:**
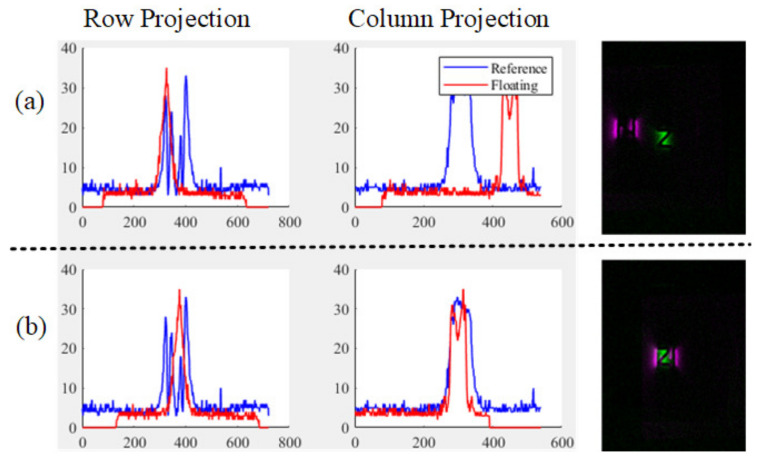
The comparison of graphs along the row and column directions; (**a**) Before transformation, (**b**) after transformation.

Because the background of the MO image is clear, it is easy to find the location of the defect. Firstly, obtain the projection along the row and column direction from the *R* and the *F*. Then, the cosine distance of the projections is minimized by adjusting the displacement along the row and column. As seen in Figure 6, the projection of the defect in two directions would partially overlap, and the cosine distance is used to assess the degree of overlap, which is calculated as

(2) $$\begin{array}{c} cos\left(\Theta \right)=\frac{Rp\cdot Fp}{\left|\left|Rp\right|\right|\cdot \left|\left|Fp\right|\right|}=\frac{\sum_{i=1}^{n}\left(Rp_{i}\cdot Fp_{i}\right)}{\sqrt{\sum_{i-1}^{n}Rp_{i}^{2}}\sqrt{\sum_{i-1}^{n}Fp_{i}^{2}}} \end{array}$$

where Rp and Fp are the vectors resulting from the projections of *R* and *F* along the row and column directions, respectively, *n* is the number of elements in Rp and Fp, and *i* is denoted as the traversal of each element.

**Figure 7:**
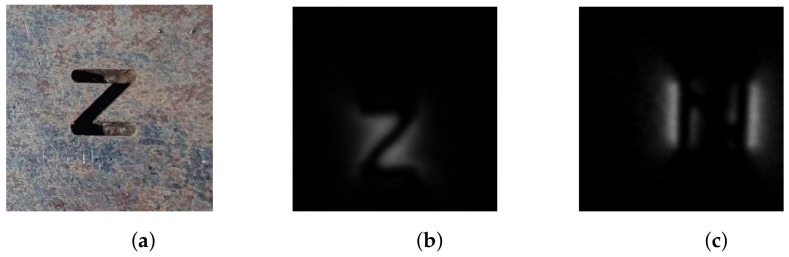
The example image images. (**a**) The original image of the defect. (**b**) The first direction of magnetization. (**c**) The second direction of magnetization (the angle between c and b is about 85${}^{\circ }$∼90${}^{\circ }$).

The displacement range in the iteration of the following optimization algorithm depends on the width of the magnetic field distribution in the image. (In the subsequent process, only the displacement part of the image will be shown; take Figure 7 as an example).

### 2.2. Segmentation

The first step is in segmenting the original images to extract the partial edge of the defects. The MFL field distribution among the edge of defects is usually sharp because the magnetic line leaks out from the edge, and the intensity decreases as the distance grows. Such a distribution could be presented by the gray intensities of the captured images. The Laplacian of the Gaussian (LoG) filter, which could be approximated by using the Difference of Gaussian (DoG), shows a good performance in identifying the edge of the defect [19,20]. The output image (O) can be calculated by:

(3) $$O\left(x,y\right)=\nabla^{2}\left(I\left(x,y\right)*G\left(x,y\right)\right)$$

where the *I* is the input image, *G* is the Gaussian filter, and $\nabla^{2}$ is the Laplacian.

**Figure 8:**
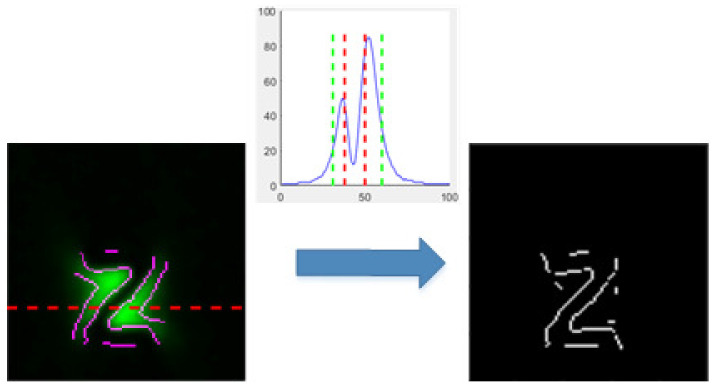
The segmentation of captured image.

However, it is easy to identify the inner and outer edges of the MFL field while detecting the border. Considering that the intensity of the MFL field is inversely proportional to the square of the distance between the defects and other parts, there has always been a peak beside the edge of defects, which can be used as a further judgment. Here, we set a limit distance that the detected point is to the peaks along the direction of magnetization. This is retained when the extracted points of LOG are within the set distance from the peak, and it is discarded if it is outside the set distance. Additionally, the result is presented in Figure 8.

### 2.3. Particle Swarm Optimization Algorithm

This study uses the PSO algorithm to update the transformation function. In PSO, each particle represents a possible solution to the optimization task, and the particles tend to cluster where the optimum solution is.

Here, the rigid geometric transformation is applied. The floating image is registered to the reference image with a global transformation. The registration parameter is a three-dimensional vector, which consists of one rotation angle θ (unit: degrees) and two translation distances $t_{x}$,$t_{y}$ (unit: millimeters). The transformation matrix of image coordinates $P_{F}$ to $P_{R}$ from the image *F* to image *R* can be shown as:

(4) $$\begin{array}{c} P_{R}=M\times P_{F}\; \end{array}$$

where

(5) $$\begin{array}{c} M=\left(\begin{array}{ccc} \cos\theta & -\sin\theta & \left(1-\cos\theta \right)t_{x}+t_{y}\times \sin\theta \\ \sin\theta & \cos\theta & \left(1-\cos\theta \right)t_{x}-t_{y}\times \sin\theta \\ 0 & 0 & 1 \end{array}\right) \end{array}$$

Therefore, the search space and the position of each particle of PSO can be represented by a three-dimensional vector $x_{i}=\left(t_{x},t_{y},\theta \right)$ [21,22]. The task performed in each particle includes:

- Transforming the image $F_{s}$.
- Constructing the shapes of defects.
- Generating the MFL field distribution ($I_{g}$) of this defect.
- Calculating the similarity between the image *R* and $I_{g}$.

The new position can be updated according to each iteration’s local and globally optimal solutions. The own personal best solution so far by the particle $p_{id}\left(t\right)$ and the global best position of any particle in the swarm $P_{gd}\left(t\right)$ so far are found. They are called the “individual best position” and the “global best position”. The new velocity and position of each particle can be updated according to $p_{id}\left(t\right)$ and $P_{gd}\left(t\right)$:

(6) $$\begin{array}{cc} \; & v_{i}\left(t+1\right)=w\left(t\right)\times v_{i}\left(t\right)+c_{1}\times \mathrm{Rand}\times \left(P_{id}\left(t\right)-x_{id}\left(t\right)\right)\\ \; & +c_{2}\times \;\mathrm{Rand}\;\times \left(P_{gd}\left(t\right)-x_{i}\left(t\right)\right) \end{array}$$

(7) $$x_{i}\left(t+1\right)=x_{i}\left(t\right)+v_{i}\left(t+1\right)$$

where $c_{1}$ and $c_{2}$ are two constants parameters. $c_{1}$ can change the step size of a particle moving toward its individual optimal position. $c_{2}$ can change the step size for moving toward the global optimal position. (Here, for a better global optimum search, $c_{2}$ is set to be slightly larger than $c_{1}$. In this study, $c_{1}$ used for an image of size 100×100 is 0.5, and $c_{2}$ is 0.7, for reference only). Rand is a random value limited by (0,1). w(t) is called the inertia weight, which influences the current velocity according to the particle’s previous velocity, and is updated in the iterations by:

(8) $$w\left(t\right)=\left(w_{\max}-w_{\min}\right)\times \frac{\left(t_{\max}-t\right)}{t_{\max}}+w_{\min}$$

where $w_{\max}$ and $w_{\min}$ denote the maximum and minimum of inertial weight, respectively. $t_{\max}$ is the maximum iteration number.

The positions of the particles should be restricted in $\left[X_{\min},X_{\max}\right]$, $\left[Y_{\min},Y_{\max}\right]$ to avoid particles escaping from the feasible solution space. Additionally, the domains are calculated in Section 2.1.

### 2.4. Opening Shape Reconstruction

After transformation, the $F_{s}$ would be partially overlaid with $R_{s}$, and then they are fused to construct the shapes of defects, which would be used to generate the distribution of the MFL field in the later process. First, two images are overlaid (performing OR operations on the corresponding pixel points) to generate $F_{temp}$, and Closing (morphology) is used to connect the adjacent connected domains, so that all of the regions in the image are connected. The Closing can be presented as:

(9) $$F_{\mathrm{temp}}•K=\left(F_{\mathrm{temp}}⊕K\right)⊖K$$

where the *K* is the structural element, and

(10) $$F_{\mathrm{temp}}⊖K=\left\{x,y|{\left(K\right)}_{xy}\subseteq F_{\mathrm{temp}\;}\right\}$$

(11) $$F_{\mathrm{temp}\;}⊕K=\left\{x,y|{\left(K\right)}_{xy}\cap F_{\mathrm{temp}}\neq \emptyset \right\}$$

The closed area would then be detected and filled, to generate the shape of the defect.

### 2.5. Solenoid Modal for the Visualization of MFL

**Figure 9:**
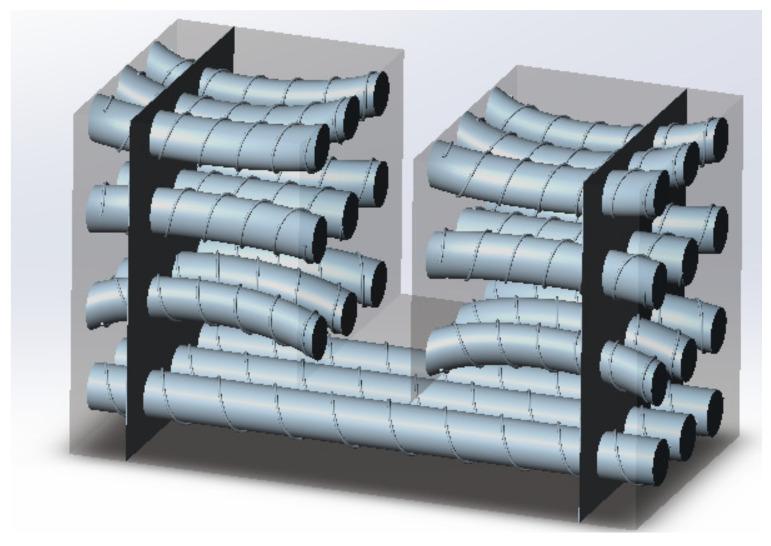
The solenoid in specimen.

The solenoid modal is adjusted to generate the distribution of the magnetic field of the constructed shape. It is based on the theory of ampere molecular currents, which are arranged neatly and closely along the magnetization direction in the specimen. Then, the molecular currents form a series of solenoids in the specimen, as shown in Figure 9.

When a solenoid is truncated, a semi-infinite solenoid model can be established to simulate the MFL on the surface of the defect, according to Biot–Savart’s law. The intensity can be calculated by:

(12) $$dH_{ls}=\frac{M_{de}}{4\pi }\frac{r_{s}}{r_{s}^{3}}ds$$

where $M_{de}$ is the effective component of $M_{d}$ in the normal direction at the defective surface, and $M_{d}$ is the magnetization. ds is an element size at the surface of the defect, and $r_{s}$ is a vector from the pole of the solenoid to the point of the field.

The above model is constructed on the assumption that the magnetization on the defect surface is uniform. However, the directions of each solenoid change due to the interaction force for actual complex defects, which makes the MFL field distribution more complicated. The magnetization of the specimen is assumed to be quasi-saturated, and the interaction force among the solenoids should be introduced to improve the model. Since the force is proportional to the magnetic field intensity, the intensity of the interaction field can be calculated by:

(13) $$\begin{array}{c} H_{\mathrm{int}er}=\frac{M_{de}}{4\pi }a\tan\left(\frac{r_{s}ds}{r_{s}^{3}}\right) \end{array}$$

The interaction between the solenoids causes the solenoids to deviate from the direction of the excitation field, and the deviation angle can be calculated by:

(14) $$\begin{array}{c} \theta =a\tan(\frac{H_{\mathrm{int}er}}{H_{ex}}) \end{array}$$

where $H_{ex}$ is the magnetic field intensity in the specimen, and here, it is set according to the material of the specimen.

**Figure 10:**
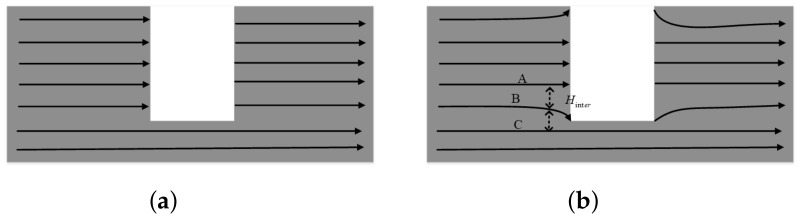
Schematic of solenoid interaction. (**a**) No interactions; (**b**) With interactions.

The interaction of the solenoids on the defect boundary is shown in Figure 10. When the interaction of the solenoids on the defect surface is not considered, the solenoids will be uniformly arranged, and their generated leakage magnetism will be uniformly distributed (Figure 10a). When the interaction between the solenoids at the defect surface is considered, the leakage magnetization generated by the solenoids at the end surface will interact with each other, resulting in the deflection of the solenoids at the end surface. In Figure 10b, we can see three solenoids, A, B, and C, at the corner of the defect. Since A and B have magnetic leakage at the end face, and C is not broken, B will be repelled by A and deflected toward C, creating a bend in B here. The influence of each solenoid at the end face by the surrounding solenoids is calculated, and this is how the solenoid model is set up. This approach has obvious advantages in the calculation of the inhomogeneous distribution of the leakage of the magnetic field at the corners of complex defects.

The applicability to the calculation accuracy of the model for complex defects is improved by modifying the direction of the solenoids. Then, the angle between the direction of the excitation field and the image should be fixed to achieve the right distribution of MFL. The depth of defects was set according to the thickness of the tested specimen and the maximal intensity of the originally captured images. Therefore, the solenoid distribution of the defective edges can be calculated. The intensity of image $I_{g}$ can be derived by the calculated magnetic field strength, and the formula is determined by:

(15) $$\begin{array}{c} I=I_{0}\sin^{2}\left(VBL\right) \end{array}$$

where $I_{0}$ is the maximal intensity of captured images.

**Figure 11:**
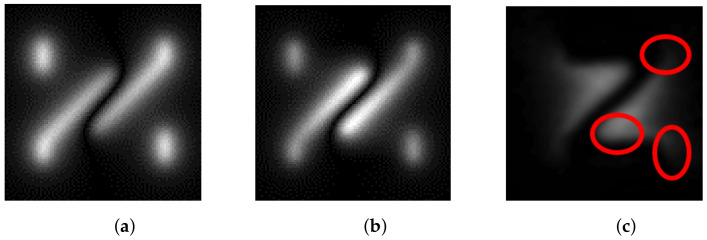
Comparison of magnetic dipole model and solenoid model. (**a**) Magnetic dipole model, (**b**) Solenoid model, (**c**) The actual MOI image.

The comparison between different methods is shown in Figure 11. Compared with the widely used magnetic dipole model (MDM), the present model has a more obvious advantage in considering the signal differences in defect corner coupling and edges (several places circled in Figure 11c have uneven signal distributions when the defect shape is complex, which cannot be fitted by MDM, while the solenoid model performs this better). Therefore, the solenoid model can better approximate the actual MFL image.

### 2.6. Similarity Measure

Now that the images (*R* and $I_{g}$) for assessing registration are ready, this section should apply a coefficient to measure the similarity between the images to evaluate the registration.

Mutual-Information (MI) is the most popular and widely studied similarity metric in intensity-based registration [21,23]. It is developed from information entropy to describe the relationship between two images. Take two random variables as an example, A and B, with marginal probability distributions, $P_{A}\left(a\right)$ and $P_{B}\left(b\right)$; joint probability distribution, $P_{AB}\left(a,b\right)$, are statistically independent if

(16) $$\begin{array}{c} P_{AB}\left(a,b\right)=P_{A}\left(a\right)•P_{B}\left(b\right) \end{array}$$

they are maximally dependent if they are related by a one-to-one mapping *T*:

(17) $$\begin{array}{c} P_{A}\left(a\right)=P_{B}\left(T\left(a\right)\right)=P_{AB}\left(a,T\left(a\right)\right) \end{array}$$

MI, I(A,B) measures the degree of dependence of A and B by measuring the distance between the joint distribution $P_{AB}\left(a,b\right)$ and the distribution associated with the case of complete independence $P_{A}\left(a\right)•P_{B}\left(b\right)$.

(18) $$\begin{array}{c} MI\left(A,B\right)=\sum_{a,b}p\left(a,b\right)\log\frac{p(a,b)}{p(a)p(b)} \end{array}$$

If the images are geometrically aligned, the MI of the image intensity values for the corresponding pixel pairs should be maximum. This criterion is very general and robust. It can be applied automatically without prior segmentation, because no assumptions about the relationship between the two image intensities are made. These properties are appropriate for calculating the similarity between the generated and original graphics, so here, we use MI as a registration criterion.

The whole algorithm of the registration of visualizing MFL testing under DOM is presented in Algorithm 1.
**Algorithm 1** Registration**Input:** Captured MFL images *R* and *F***Output:** The optimal registration parameters $\left(\theta ,t_{x},t_{y}\right)$; 1: Segmeting the images to $R_{s}$ and $F_{s}$; 2: Setting the particles and iterations number of PSO; 3: **repeat** 4:    Updating a new registration parameter; 5:    Transforming the $F_{s}$; 6:    Reconstructing a shape of crack ($I_{t}$); 7:    Generating a new distribution ($I_{g}$) of ($I_{t}$); 8:    Calculating the similarity between *R* and $I_{g}$; 9:    Recording the global and local optimal positions.10: **until** The iteration of PSO finishes.

**Figure 12:**
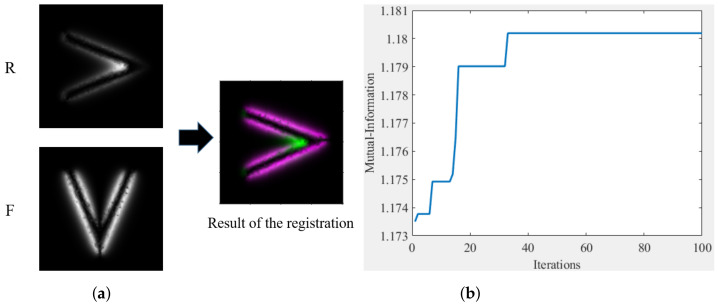
Example of operational feasibility. (**a**) Results of registration, (**b**) Convergence of iterations.

Based on the method designed above, we take the first two simulated images in Figure 1 with the first two magnetization directions differing by 90° as an example; the image size is 100×100, and the relative displacement of the two images in the row and column directions is 0 pixels. The number of particles in the optimization algorithm is set to 5, and the number of iterations is set to 100. We can obtain the registration results, as shown in Figure 12a. The convergence process of the iterations is shown in Figure 12b, which shows that the similarity reaches its best after 33 iterations, with a similarity of 1.1802. The registration result is a rotation angle of 92.0510°, with a displacement of 0.46805 pixels along the row direction, and −0.64315 pixels along the column direction. This registration result has some errors with the actual results, but it shows that the method is iterative convergent.

## 3. Experiment Setup

### 3.1. The Settings of the Experimental Equipment

**Figure 13:**
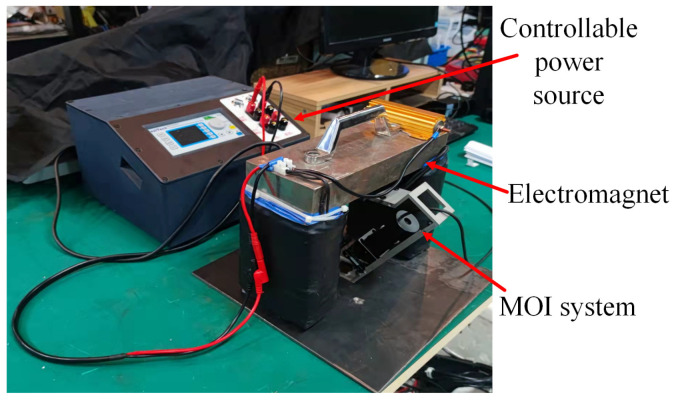
The platform of MFL image detection.

In order to verify the proposed methods, the experimental platform is set up (shown in Figure 13). The electric magnetic yoke is the magnetizing excitation source to generate a uniform excitation magnetic field. The MOI system is fixed in the middle of the magnetic yoke, making the angle between the excitation direction and the captured image constant.

Additionally, the MO film is a ${\left(BiTm\right)}_{3}{\left(GaFe\right)}_{5}O_{12}$ single-crystal thin film [24,25] provided by the State Key Laboratory of Electronic Thin Films and Integrated Devices, University of Electronic Science and Technology of China, and the maximum Verdet constant $2.595\times 10^{-4}/\left(Oe\mu m\right)\cdot 10\;\mu m$.

### 3.2. Experiment Samples

**Figure 14:**
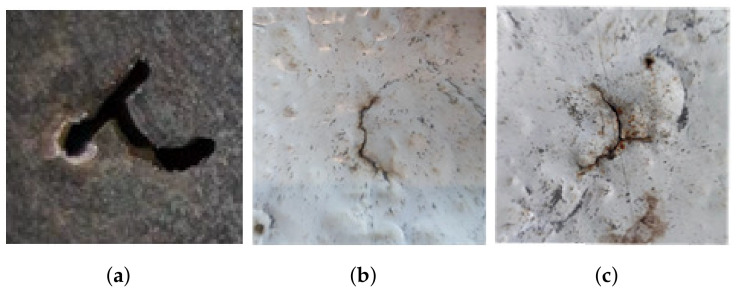
The samples: (**a**) Manual t-shaped crack, (**b**) Natural one-line crack, (**c**) natural three-line crack.

The test samples include two artificial and two natural cracks. The artificial samples include a z-shaped (Figure 7a) and t-shaped cracks (Figure 14a). The natural sample includes two fatigue cracks, the first is a crack with one-line (Figure 14b), and the other one is a crack with three-line coupling (Figure 14c).

## 4. Results and Discussion

### 4.1. Experiment Results

The images in Figure 7 are used to verify the method, and the result is shown in Table 1. The variation of the parameter of the transformation matrix leads to different shapes of defects, therefore generating different distributions of the MFL field. As can be seen from the last column of Table 1, when the transformation matrix comes to the correct position, the MI similarity of the generated image ($I_{g}$) and the reference image (*R*) is optimal. We can also find that the constructed shape is the most similar to the shape of the actual defect when the similarity is optimal, which can be used to evaluate the defect. The MI similarity here shows a significant difference between the incorrect and correct registrations, and the better it is registered, the bigger the similarity coefficient is. Because of the difference in the shape of the fused defects, the magnetic field distribution deduced from the defects will also have significant differences. Meanwhile, we can see that the reconstructed shape is closest to the actual defect morphology when in the correct alignment state, and the MFL distribution calculated by the solenoid model has the highest similarity to the original image, which also proves the correctness of the solenoid model for the defect MFL calculation at the same time. Therefore, the present method can also be regarded as a reconstruction of the defect surface profile by fusing multiple sources of the MFL image data.

However, some significant factors also affect the result of the calculation of similarity between images.

1. The segmenting method for the original images *R* and *F* is significant. The better the edge of defects is detected, the better the shape of defects is constructed.
2. The fusion of segmented images and the method of shape construction directly affects the MFL field distribution of $I_{g}$.
3. The stability and accuracy of the MFL distribution forward model.

### 4.2. Robustness of the Proposed Method

The robustness of the method can be evaluated through comparisons with the feature-based, intensity-based, and manual registration methods (shown in Table 2).

The feature-based approach requires the presence of features: such as corners, outlines, and some particular points. Because of the smooth edge and the distortion, it is challenging to extract valid corresponding feature points. At the same time, the obtained features cannot be matched with each other because the magnetic field distribution generated by different magnetization directions cannot correspond to each other. All these problems make it impossible to register the images correctly.

The deviations in the magnetic field distribution also affect the intensity-based registration. The images are only matched according to intensity because the shapes of defects and magnetic field distortions cannot be considered. In the registration, calculating the intensity similarity optimum only leads to the match of the regions in two images that are close in intensity, which does not contribute to the correct registration of the images.

Manual registration shows a good performance because it is based on the existing knowledge of the shapes of the defects and the relative orientations of the images. However, manual registration cannot be employed in inspection applications with large data and for defects of unknown shape and location. This is why a method is needed for the scenarios described in this paper. In this set of comparisons, manual registration is only used as a standard group to evaluate the results of the registration of images.

The last row of Table 2 shows the experimental results of the proposed method, which can achieve a better result compared to feature-based and intensity-based registration methods, and it is more consistent with the manual one. For cracks, it can achieve a high registration accuracy due to the prominent structural characteristics of the magnetic field distribution, and the error of the crack registration mainly occurs in the translation. The error in defect reconstruction and the difference between the actual data and the model derivation make a certain deviation in the registration results, which needs to be enhanced and solved in the subsequent study.

Here, we use the correlation coefficient to calculate the registered *F* for comparison with the manually registered *F* to evaluate the registration results of different methods. Equation (19) is the calculation of the correlation coefficient:

(19) $$\begin{array}{c} Cor=\frac{\sum_{i=1}^{N}\left(Fm_{i}-\bar{Fm}\right)\cdot \left(Fa_{i}-\bar{Fa}\right)}{\sqrt{\sum_{i=1}^{N}{\left(Fm_{i}-\bar{Fm}\right)}^{2}\cdot \sum_{i=1}^{N}{\left(Fa_{i}-\bar{Fa}\right)}^{2}}}\cdot 100\% \end{array}$$

where Fm is the manually registered *F* and Fa is the *F* registered using other methods. *N* denotes all the pixels in the graph, and *i* denotes the transversal of each element of it. The closer the Cor is to 100%, the closer is the result of the registration method used to the manual registration result.

**Figure 15:**
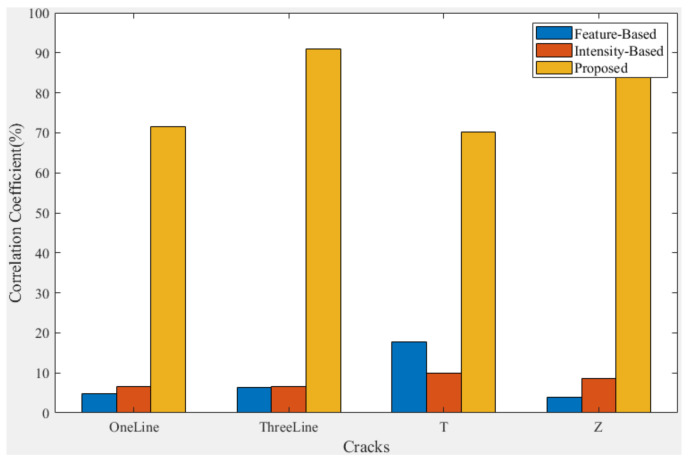
Comparison of correlation coefficients with manual registration results.

As shown in Figure 15, by comparing the registration results of different methods with the manual registration results, it can be seen that the feature-based and intensity-based methods are less similar to the results of the manual registration methods, and they can even be considered to be completely unusable. Additionally, the proposed method has a clear advantage in that it can have better registration results for all of the cracks tested here.

It can be observed from the above results that the proposed method can be used for the MFL information fusion of cracks under DOM, which helps in the subsequent analysis of defects.

## 5. Conclusions

This study presented a registration method for MFL field visualization under DOM. It solved the problem of the mismatch between the distorted images captured under DOM by considering the 2D shape reconstruction of defects, and the application of the forward model. The solenoid model for MFL visualization is first applied to analyze and to generate the MFL field distribution. The experimental results showed that the proposed method performs better in MFL image registration than do the currently used methods. The proposed method can be applied in MFL detection engineering applications for crack detection. Future research could focus on image segmentation, shape reconstruction, and improving the calculation accuracy of the forward model. Additionally, it could be noticed that such a registration structure could also be used in multimodel situations, such as registering images captured using different NDT methods.

## Figures and Tables

**Table 1:** The similarity between the $I_{g}$ and *R* changes according to the deviation of $F_{s}$.

–	Angle + $30^{\circ }$	X + 10	Y + 10	Correct
$R_{s}$	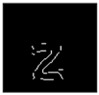	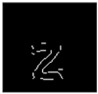	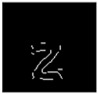	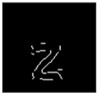
$F_{s}$	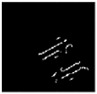	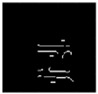	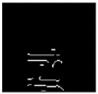	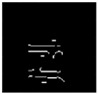
$F_{temp}$	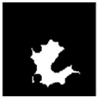	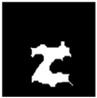	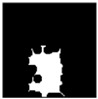	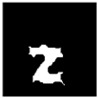
$I_{g}$	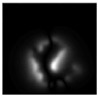	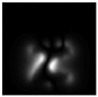	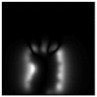	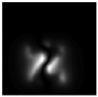
Similarity	0.6446	0.7503	0.6589	0.9200

**Table 2:** The result of image registration from different methods. (The feature-based and intensity-based results come from MATLAB 2019b image registration app: the surf and multimodal intensity module).

–	T	Z	OneLine	ThreeLine
*R*	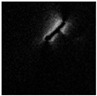	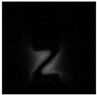	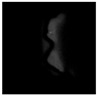	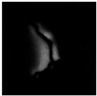
*F*	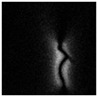	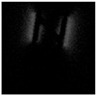	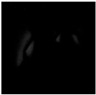	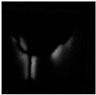
Feature-Based	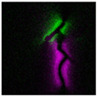	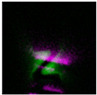	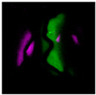	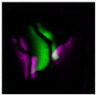
Intensity-Based	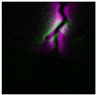	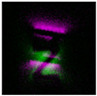	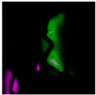	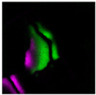
Manual	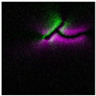	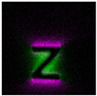	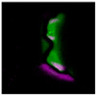	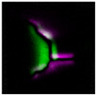
Proposed	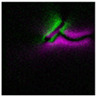	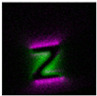	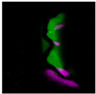	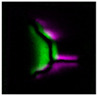
